# Genome Sequences of Three African Swine Fever Viruses of Genotypes I, III, and XXII from South Africa and Zambia, Isolated from *Ornithodoros* Soft Ticks

**DOI:** 10.1128/MRA.01376-19

**Published:** 2020-03-05

**Authors:** S. Ndlovu, A.-L. Williamson, R. Malesa, J. van Heerden, C. I. Boshoff, A. D. S. Bastos, L. Heath, O. Carulei

**Affiliations:** a Division of Medical Virology, Department of Pathology, Faculty of Health Sciences, University of Cape Town, Cape Town, South Africa; b Agricultural Research Council-Onderstepoort Veterinary Research, Cape Town, South Africa; c Institute of Infectious Diseases and Molecular Medicine, Faculty of Health Sciences, University of Cape Town, Cape Town, South Africa; d Tshwane University of Technology, Department of Biomedical Sciences, Pretoria, South Africa; e University of Pretoria, Department of Zoology & Entomology, Pretoria, South Africa; DOE Joint Genome Institute

## Abstract

Here, we report the draft genome sequences of three African swine fever viruses isolated from *Ornithodoros* soft ticks. Isolates LIV 5/40 (Zambia), SPEC 57 (South Africa), and RSA/2/2008 (South Africa) belong to genotypes I, III, and XXII, respectively.

## ANNOUNCEMENT

African swine fever (ASF) is a hemorrhagic disease affecting domestic pigs that has mortality rates approaching 100%. African swine fever virus (ASFV) is the causative agent of ASF and is the only species belonging to the *Asfivirus* genus of the *Asfarviridae* family. In sub-Saharan Africa, ticks of the genus *Ornithodoros* are the main reservoir of ASFV (1, 2). ASFV has a linear double-stranded DNA genome ranging from 170 to 190 kbp in length, encoding 160 to 167 open reading frames (ORFs). It is the sole member of the *Asfarviridae* family, genus *Asfivirus*, and the only known DNA arthropod-borne virus (arbovirus) (3). To date, 24 genotypes have been described based on C-terminal *p72* gene sequencing (4–8). However, publicly available, complete genome sequences represent only genotypes I through V, VII through X, and XX, with the vast majority of sequences corresponding to genotypes I, II, and IX (9–13), thus limiting the understanding of the geographical origin and outbreak patterns. Viral DNA was extracted from primary pig macrophages infected with homogenates of whole ticks collected in Zambia in 1983 (LIV 5/40), South Africa in 2008 (RSA/2/2008), and South Africa in 1985 (SPEC 57) using the High Pure PCR template preparation kit (Roche Diagnostics, Germany). The NEBNext microbiome DNA enrichment kit (New England BioLabs, USA) was used to deplete host-methylated DNA. The isolated DNA was amplified with the Genomiphi V2 DNA amplification kit (GE Healthcare, USA), and conventional PCR amplification and sequencing of the C-terminal *p72* gene region were used to confirm ASFV nucleic acid presence and genotype, respectively, as described previously (4). Genome sequencing was performed on an Illumina HiSeq sequencer using the Nextera XT DNA sample preparation kit and the V2 reagent kit for 2 × 250-bp paired-end reads (Illumina, USA). A *de novo* assembly of all reads was performed using CLC Genomics Workbench version 9.0.1 (Qiagen). Complete genomic contigs were generated from 2,234,811 of 22,993,178 reads, 7,322,880 of 9,914,962 reads, and 3,487,567 of 36,764,148 reads for LIV 5/40, RSA/2/2008, and SPEC 57, respectively. The remaining smaller contigs and nonspecific reads were identified as belonging to the pig genome. GATU (14) was used to annotate the genomes using BA71V (9) as the reference, CLC Genomics Workbench was used to search for ORFs in regions that were missed by GATU, and a BLAST search was performed against the NCBI database to confirm the ORFs. Totals of 164, 167, and 165 ORFs were identified for LIV 5/40, RSA/2/2008, and SPEC 57, respectively. All ORFs intact at their 5′ end but comprising less than 80% of ortholog length are annotated as truncated genes, while those ORFs disrupted at their 5′ end are annotated as fragmented genes (15).

The LIV 5/40, RSA/2/2008, and SPEC 57 isolates each had G+C contents of 38.6%, with average coverages of 3,048×, 9,623×, and 4,685× and genome lengths of 183,291 bp, 190,242 bp, and 186,118 bp, respectively. The genomic termini were not sequenced. Multiple sequence alignment and phylogenetic analysis of the C terminus of the *B646L* gene showed that LIV 5/40 belongs to genotype I, RSA/2/2008 to genotype XXII, and SPEC 57 to genotype III, with LIV 5/40 showing closest BLASTn identity to LIV/1217 from Zambia (genotype I; GenBank accession number AY351524), RSA/2/2008 to RSA/1/2002 from South Africa (genotype XXII; JX403678), and SPEC 57 to RSA/3/2003 from South Africa (genotype III; JX403655). A BLASTn search against the NCBI database with default parameters showed that the genome sequences most similar to the LIV 5/40, RSA/2/2008, and SPEC 57 genomes were those of strain L60 (KM262844; genotype I; 95% identity and 93% coverage), isolate Warthog (AY261366; genotype IV; 98% identity and 95% coverage), and isolate Pretorisuskop/96/4 (AY261363; genotype XX; 97% identity and 93% coverage), respectively. These data add to the pool of diverse ASFV isolates available for comparative genomics studies.

### Data availability.

The ASFV genome sequences in this report are available in GenBank under accession numbers MN318203 (LIV 5/40), MN336500 (RSA/2/2008), and MN394630 (SPEC 57). The raw reads are available in the SRA under accession numbers PRJNA577538 (LIV 5/40), PRJNA577445 (RSA/2/2008), and PRJNA577546 (SPEC 57).
